# Congenital Talipes Equino-Varus

**Published:** 1907-08-24

**Authors:** 


### CONGENITAL TALIPES EQUINO-VARUS.

### I.—GENERAL CONSIDERATIONS.

We have little exact knowledge of the causation of congenital talipes equino-varus. It is generally attributed to mechanical causes due to malposition of the fœtus in utero. According to Parker, who has given special attention to the subject, there is commonly a small degree of equino-varus even in normal infants, and the tendency to inversion of the foot is subsequently lost when they begin to walk. If the tendency to inversion is advanced, the malposition is not corrected, and the deformity is permanent. He states that the common modes of production of the intra-uterine deformity are as follows: (1) Locking of the parts due to abnormal position of the limbs; (2) abnormal position of the limbs without locking; (3) abnormal position of the limbs due to intra-uterine pressure from deficiency of the liquor amnii. In addition to these, a small proportion of cases are undoubtedly due to imperfect osseous development or to spinal anomalies, particularly to spina bifida.

From an anatomical point of view the deformity is a double one; there is an abnormal condition of the parts at the ankle as well as at the mid-tarsal joint. Clinically the condition may be recognised by the following points: (1) The heel is drawn up and the foot is extended; the heel itself is often imperfectly developed. (2) The whole foot, anterior to the mid-tarsal joint, is drawn inwards, and is rotated at the joint so that the sole is inverted. (3) The internal border of the foot is shortened and bent upon itself, so that the inner malleolus is buried in its concavity. (4) The external border of the foot is convex; and after the child has begun to walk callosities are developed in the skin over it because the weight of the body is transmitted to this part. (5) The upper surface of the astragalus forms a projection on the dorsum of the foot.

Cases of talipes may be arbitrarily divided into three classes. The first is that in which the deformity can be rectified by manipulation. It is rare to meet cases in quite young infants where the foot cannot be brought into position by the exercise of slight manual force. Occasionally the abnormal shape of the bones is so marked as to render this impossible. But even where this is the case, treatment by manipulation, if patiently carried out, will eventually be successful, since the bones are still cartilaginous, and can be moulded in a surprising manner.

The second class of case is met with in children who have begun to walk without any attempt having been made to treat the condition. Here restitution of the foot to the normal position is resisted by the shortening and contraction of the soft parts. The ligaments affected are the plantar fascia, the anterior part of the deltoid ligament, and those surrounding the astragalo-scaphoid articulation, particularly the spring ligament. The muscles of the affected limb are ill-developed, and do not acquire the same size as those of the normal limb. The shortening of the tendo Achillis tends to increase the extension at the ankle-joint, and the contraction of the tibialis posticus increases the inversion of the sole of the foot owing to its intimate association with the bones of the tarsus. The short muscles of the sole of the foot are also secondarily contracted. In these cases the deformity can only be corrected after tenotomy of the resisting structures.

The last class consists of cases of talipes in adults, in whom the bones have been allowed to ossify in their abnormal position. The neck of the astragalus is now permanently deflected downwards and inwards, so that its obliquity is greatly increased, and its upper articular surface is so much broadened that it cannot be replaced between the malleoli, and the extension at the ankle-joint cannot be reduced without an open operation. Furthermore, ankylosis has taken place at one or more of the normal tarsal joints, and new ones have been formed to meet the altered condition of affairs.

In dealing with any case of talipes, the practitioner must be prepared to answer two questions which the relatives of such patients always ask:

1. Will the patient ultimately have a shapely foot?
2. Will he be able to walk as well as a normal individual?

In an infant who has not yet begun to walk the result of treatment begun at once and continued patiently over a long period is almost uniformly good: a fact which accounts for the comparative rarity of cases of bad talipes in adults of the well-to-do classes. But when secondary contraction of the soft parts has already occurred, or when the bony changes have been allowed to become permanent, a guarded prognosis must be given, because the results of such cases are proportionately disappointing.

